# Supplementary material for: Input dependent modulation of olfactory bulb activity by HDB GABAergic projections
Source: Sci Rep. 2020 Jul 1;10:10696. doi: 10.1038/s41598-020-67276-z (PMC7329849; doi:10.1038/s41598-020-67276-z)
Supplement: Supplementary file 1 — Supplementary Information. [file 41598_2020_67276_MOESM1_ESM.docx]

**Supplementary Information**

**Input dependent modulation of olfactory bulb activity by HDB GABAergic projections**

Erik Böhm^1^, Daniela Brunert^1^, Markus Rothermel^1*^

^1^ Department of Chemosensation, AG Neuromodulation, Institute for Biology II, RWTH Aachen University, Aachen 52074, Germany

* Correspondence:

Dr. Markus Rothermel

Institute for Biology II – Dept. Chemosensation – AG Neuromodulation

RWTH Aachen University

2. Sammelbau Biologie, Room 2.111

Worringerweg 3

D-52074 Aachen

Germany

Phone: +49 241 80-20831

Fax: +49 241 80-22133

m.rothermel@sensorik.rwth-aachen.de

Supplementary Figures S1-4

**
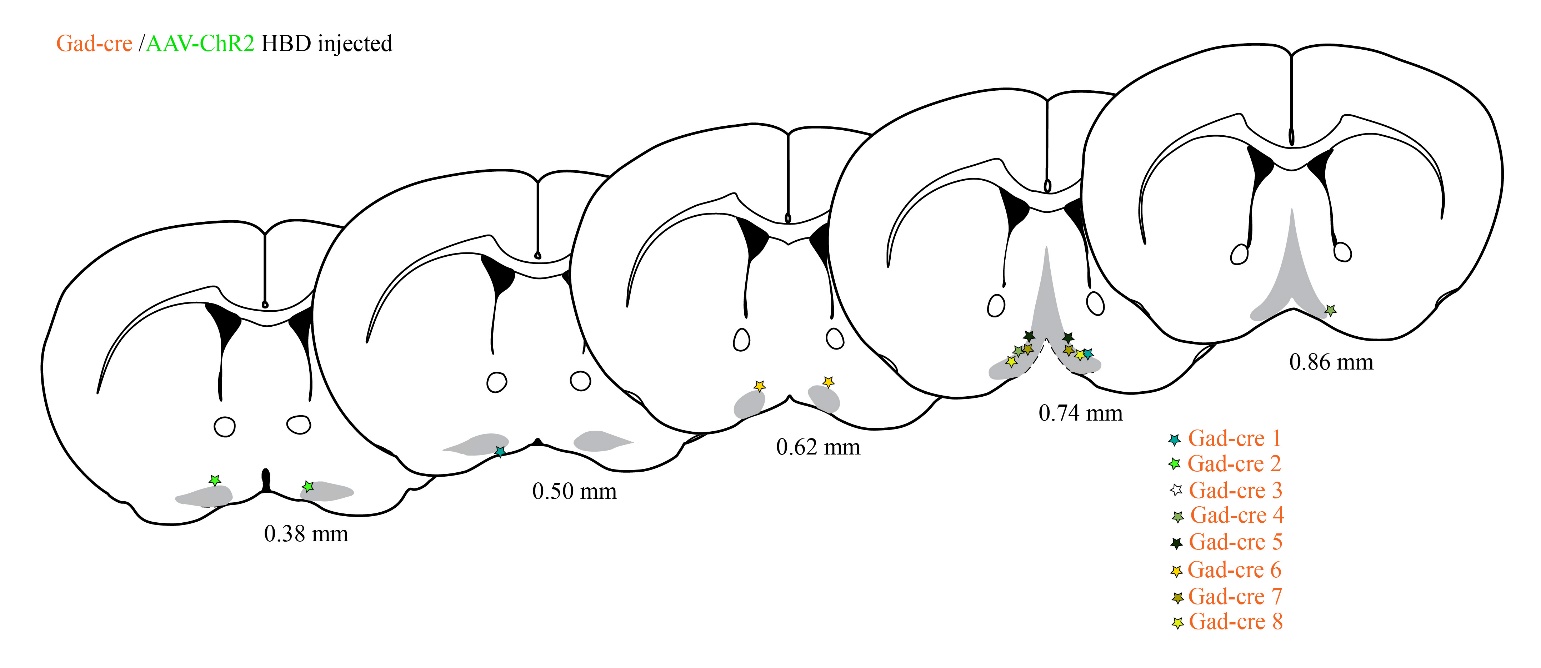
Supplemental Figure 1. Histological reconstruction of basal forebrain injection sites in GAD-Cre mice**

Schematic illustration of the reconstructed injection sites from eight GAD-Cre animals injected with AAV-ChR2. HDB/VDB BF area is marked in grey. Bregma values from ^1^.

**
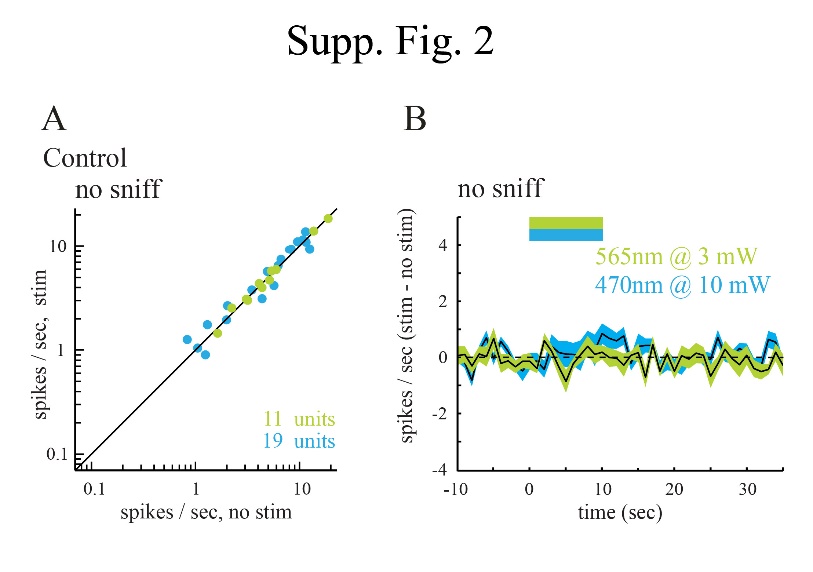
Supplemental Figure 2. Light-evoked modulation of spontaneous MTC firing in ChAT-Cre and GAD-Cre mice is attributable to cholinergic/GABAergic signaling**

A. Plot of the spontaneous firing rate of MTCs in control, uninjected ChAT-Cre or GAD-Cre mice before and during optical stimulation of the OB (n = 11 units, 470nm @ 10 mW, blue; n = 19 units 565 nm @ 3 mW, green), recorded and analyzed as in Fig 2B.

B. Time course of firing rate changes across all recorded MTCs during optical stimulation in control mice.

**
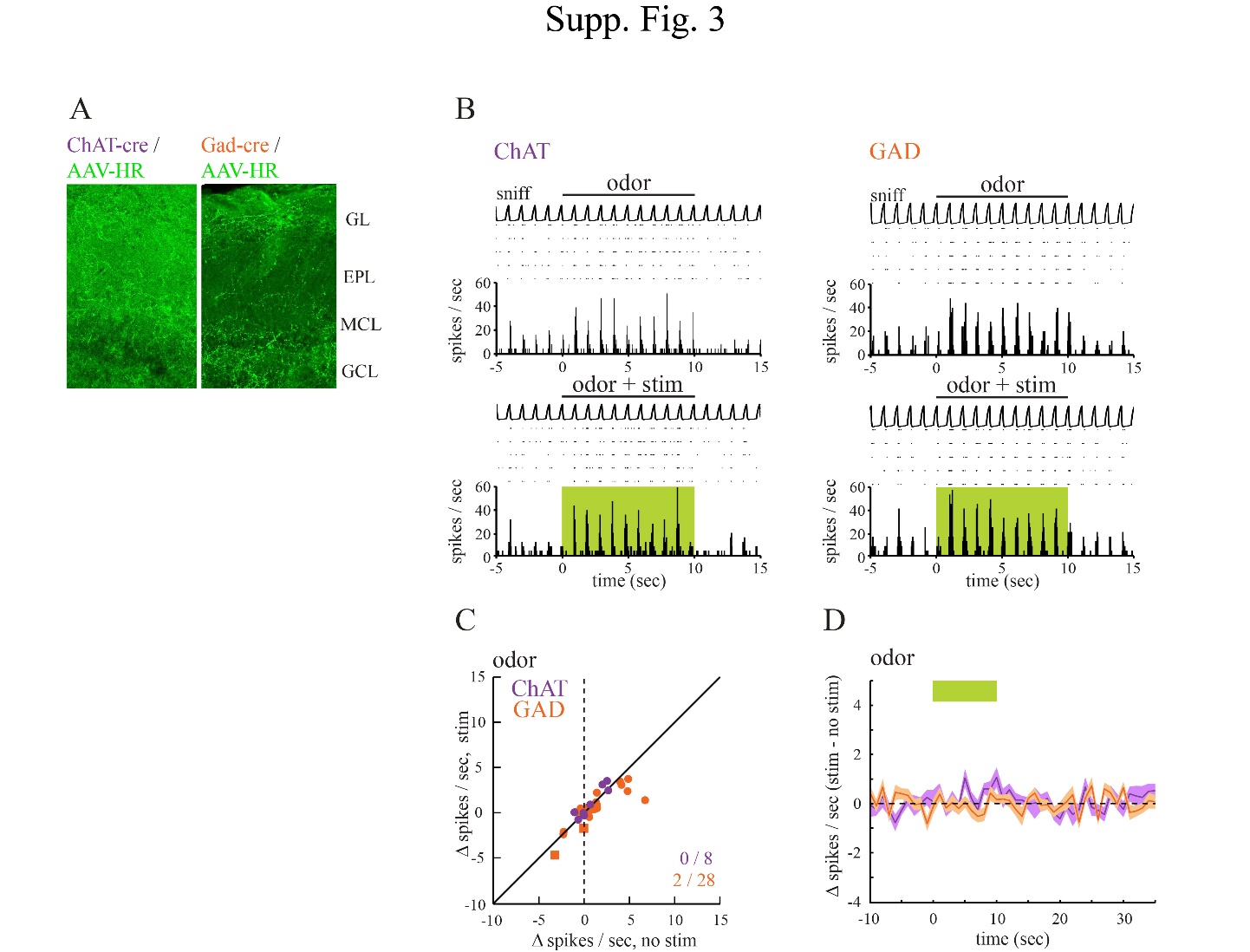
**

**Supplemental Figure 3. Effects of optogenetic silencing of cholinergic and GABAergic OB inputs on MTC odor-evoked responses.**

A. HR-EYFP-expressing axon terminals in the OB imaged 4 weeks after AAV-HR-EYFP injection into BF of ChAT-Cre and GAD-Cre mice. GL: glomerular layer, EPL: external plexiform layer, MCL: mitral cell layer, GCL: granule cell layer.

B. Odorant-evoked MTC spiking in response to optical OB silencing in ChAT-Cre and GAD-Cre mice.

C. Plot of odorant-evoked changes in MTC spiking (∆ spikes/sniff) in the absence of (no stim) and during (stim) optogenetic silencing of cholinergic (n = 8 units, purple) or GABAerig (n = 28, orange) afferents to the OB.

D. Time course of effects of optical silencing on odorant-evoked spike rate, averaged across all units. The green bar shows the time of optical stimulation and simultaneous odorant presentation. The shaded area indicates variance (SEM) around mean.

**
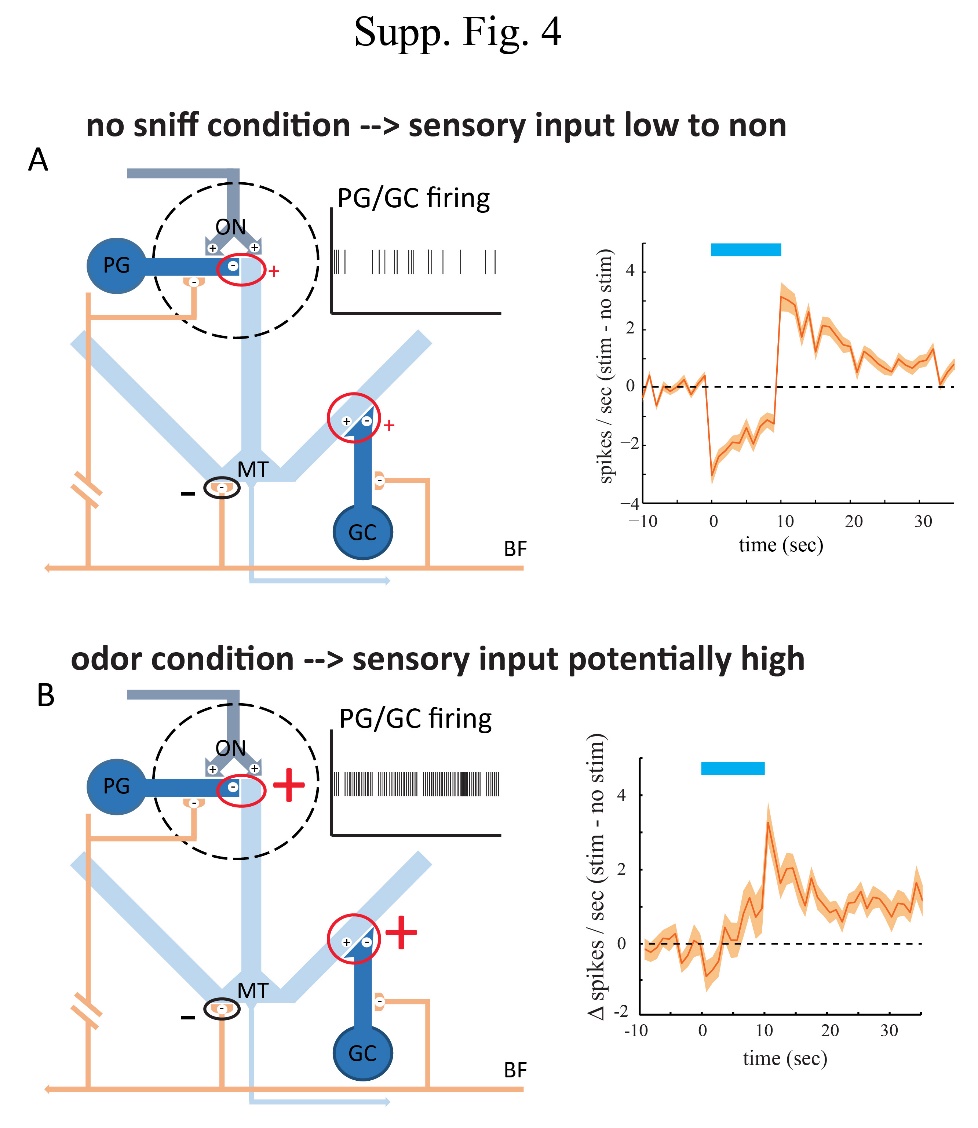
Supplemental Figure 4. Possible mechanism of input dependent GABAergic modulation of MTCs.**

Schematic diagram summarizing the presumed connectivity of the GABAergic projection from basal forebrain (adapted from ^2^, GC= granule cells PG= periglomerular cells, ON= olfactory nerve, MT= mitral/tufted cells).

A: In the no sniff condition, with little to no sensory input, inhibitory interneurons like PGs and GCs show little activity. This means that GABAergic BF derived inhibition of these cells has little effect and little to none disinhibition of MTCs can be observed. At the same time, direct inhibition of BF derived fibers on MTCs might mediate the inhibitory effect seen in this condition (right).

B: In the odor condition, with strong sensory input, inhibitory interneurons like PGs and GCs show stronger activity. Therefore, GABAergic BF derived inhibition of these cells can lead to the disinhibition of MTCs potentially outweighing the direct inhibition of MTCs.

1 Paxinos, G. & Franklin, K. B. J. *The Mouse Brain in Stereotaxic Coordinates*. Second Edition edn, (Academic Press, 2001).

2 Gracia-Llanes, F. J. *et al.* GABAergic basal forebrain afferents innervate selectively GABAergic targets in the main olfactory bulb. *Neuroscience* **170**, 913-922 (2010).
